# Immunotherapy response evaluation with magnetic resonance elastography (MRE) in advanced HCC

**DOI:** 10.1186/s40425-019-0766-y

**Published:** 2019-11-28

**Authors:** Aliya Qayyum, Ken-Pin Hwang, Jason Stafford, Anuj Verma, Dipen M. Maru, Subramanya Sandesh, Jia Sun, Roberto Carmagnani Pestana, Rony Avritscher, Manal M. Hassan, Hesham Amin, Asif Rashid, Ignacio I. Wistuba, Richard L. Ehman, Jingfei Ma, Ahmed O. Kaseb

**Affiliations:** 1 Department of Abdominal Imaging, UT MD Anderson Cancer Center, 1400 Pressler Street, Houston, Texas USA; 2 Department of Imaging Physics, UT MD Anderson Cancer Center, Houston, Texas USA; 3 Department of Translational Molecular Pathology, UT MD Anderson Cancer Center, Houston, Texas USA; 4 Department of Pathology, UT MD Anderson Cancer Center, Houston, Texas USA; 5 Department of Biostatstistics, UT MD Anderson Cancer Center, Houston, Texas USA; 6 Department of GI Medical Oncology, UT MD Anderson Cancer Center, Houston, USA; 7 Department of Interventional Radiology, UT MD Anderson Cancer Center, Houston, Texas USA; 8 Department of Epidemiology, UT MD Anderson Cancer Center, Houston, Texas USA; 9 Department of Hemopathology, UT MD Anderson Cancer Center, Houston, Texas USA; 10 Department of Radiology, Mayo Clinic, Rochester, Minnesota USA

**Keywords:** Hepatocellular carcinoma, Magnetic resonance Elastopgraphy, Immunotherapy

## Abstract

**Background:**

Currently, there are no imaging predictors of immunotherapy outcome in hepatocellular carcinoma (HCC). The study aim was to determine if stiffness changes measured by magnetic resonance elastography (MRE) can be a predictor of immunotherapy response in patients with advanced HCC.

**Materials and methods:**

This was a prospective study of 15 patients with biopsy proven-advanced HCC treated with Pembrolizumab. All patients had liver MRE and liver biopsy at baseline and at 6 weeks of therapy. Change in HCC stiffness on MRE was compared with overall survival (OS), time to disease progression (TTP), and number of intratumoral CD3+ T lymphocytes. Analysis was performed using descriptive statistics and Spearman correlation (*R*); *p-value* < 0.05 was considered statistically significant.

**Results:**

Nine patients were evaluable. Median age was 71 years (range, 54–78). Etiology of liver disease was HCV (*n* = 4), HBV (*n* = 1) and NASH (*n* = 4). Median OS and TTP were 44 weeks and 13 weeks, respectively. Average baseline HCC stiffness and change in HCC stiffness were 5.0 kPa and 0.12 kPa, respectively. In contrast, average non-tumor liver stiffness was 3.2 kPa, and did not significantly change at 6 weeks (*p* = 0.42). Average size of measured tumor and change in size were 4 cm and − 0.32 cm, respectively. Change in HCC stiffness at 6 weeks correlated significantly with OS (*R* = 0.81), and TTP (*R* = 0.88,*p* < 0.01). Abundance of intratumoral T lymphocytes on tumor biopsy correlated significantly with HCC stiffness (*R* = 0.79,*p* = 0.007).

**Conclusion:**

Our pilot MRE data suggests early change in tumor stiffness may be an indicator of immunotherapy response in patients with advanced HCC.

## Introduction

HCC is considered the fifth most common malignancy worldwide, with the third-highest mortality [1]. An estimated that 80% of patients present with advanced stage tumor not amenable to curative therapy [1, 2]. Oral tyrosine kinase inhibitor (sorafenib) has been the frontline standard of care since 2007 for treatment of advanced HCC with preserved liver function [3]. Newer systemic treatments with immunotherapy agents are being investigated, such as Nivolumab and Pembrolizumab (anti–PD-1 mAb) which enhance immune function and cytotoxic T-lymphocyte (CTL)-mediated immune response against cancer cells [4–6].

Imaging assessment of HCC response to targeted therapies is challenging since reduction in size may not occur. Tumor stability is used as a marker of response without necessarily conferring improved outcomes [7–9]. MR Elastography (MRE) is a relatively novel technique, and has been shown to be superior to ultrasound-based transient elastography for assessment of liver fibrosis [10]. MRE may be used to distinguish malignant from benign liver tumors [11], which is thought be due to the abnormal cellular microenvironment of neoplastic conditions, including denser extracellular matrix, increase cellularity, vascularity, and interstitial pressure, causing increased stiffness.

Immunotherapy response decreases viable tumor cells, but increases immune content, and causes stromal and fibrosis flux due to effects on immune cell function. We hypothesize that such changes in tumor cellularity and stroma in patients treated with anti-PD-1 immunotherapy would effect MRE tumor stiffness. The purpose of our study was to determine if stiffness changes measured by magnetic resonance elastography (MRE) can be a predictor of immunotherapy response in patients with advanced HCC.

## Materials and methods

This was a prospective, Institutional Review Board approved study. A total of 15 patients were accrued through our Liver Center, with biopsy proven advanced HCC (not amenable to curative therapy), Child-Pugh Score A, who were treated with anti-PD-1, Pembrolizumab monotherapy. Clinical responses were determined by blinded independent review using RECIST 1.1 and mRECIST 1.1. All patients provided written informed consent. Eligible patients were over 18 years of age with radiographic disease progression on sorafenib or intolerance to sorafenib treatment, and ECOG 0 or 1. All patients underwent liver MRI with MR Elastography (MRE) and liver biopsy at baseline and at 6 weeks of therapy. Date of documented disease progression on patient follow-up and date of death were obtained from the patients’ electronic medical records.

MR Elastography (MRE) was performed on a 3 T whole body MRI scanner (Discovery 750 HD; GE Healthcare, Waukesha, WI) with a 32-channel phased-array torso coil. Acoustic waves at 60 Hz were generated by an active driver and transmitted to the liver through an external passive driver placed over the subject’s right upper quadrant (overlying the liver). Data was acquired using a 2D-echoplanar imaging (EPI) based MRE sequence with the following imaging parameters: TR/TE = 600 ms/Minimum Full; slice thickness/gap =7/2.5 mm; FOV 38-42 cm; acquisition matrix = 64 × 64; NEX = 2; 6 axial slices through widest cross-section of the liver including at least one slice through the tumor; parallel imaging factor = 2, and acquisition time = 16–19 s (one breath hold). Automated in-line post-processing was used to generate quantitative maps or “elastograms” of liver stiffness in units of kilopascals (kPa) [12–16], and a mask overlay to exclude pixels with low inversion processing confidence [12].

### Image analysis

Liver and tumor stiffness (kPa) was measured by an independent reader blinded to pathologic and clinial data. MRE derived average non-tumorous liver stiffness (kPa) was measured by placing regions-of-interest (ROIs) on the MRE elastograms (stiffness maps) to include as much of the non-tumorous liver as possible away from the HCC. Measurements were obtained at 3 axial levels through widest portions of liver while excluding any visible major vessels [12–16]. Average HCC stiffness was measured by drawing ROIs on the elastograms covering as much of the HCC as possible. HCC size and extent of tumor enhancement were also recorded. HCC enhancement was categorized on arterial phase images using a 4-point scale (0–3), with no enhancement as 0, < 25% as 1, 25–50% as 2 and > 50% as 3.

### Pathology analysis

All subjects underwent image guided liver biopsy at baseline and at 6 weeks of pembrolizumab treatment. Histopathology analysis was performed by a pathologist in five intratumoral areas using 660 μm × 500 μm (0.33mm^2^) region of interest (ROI) at × 20 magnification to cover a total intratumoral area of 1.65mm^2^. A pancytokeratin (AE1/AE3) marker was used and the intratumoral area compartmentalized in epithelial (tumor) and stroma compartment. Total intratumoral lymphocytes (CD3^+^) was expressed as an average of cell densities from the areas analyzed (n/mm^2^). HCC tumor grade and number of T lymphocytes (CD3^+^; n/mm2) were obtained from the database of the parent MDACC-sponsored clinical protocol supported by Merck & Co.

### Statistical analysis

Summary of demographic and clinical characteristics were provided in percentages, means, medians, standard deviations (SD), and range. Spearman rank correlation was estimated between imaging measurements at the 2 time-points, and between imaging measurements and lymphocytes (n/mm2) on pathology. Overall survival (OS) or time-to-progression (TTP) was correlated with baseline and change in both HCC size and stiffness, and baseline non-tumorous liver stiffness. Comparison of tumor grade with HCC stiffness, and of changes in HCC stiffness between groups with survival of either more than or less than 52 weeks was performed using Wilcoxon rank-sum test.

HCC stiffness was correlated with HCC size at baseline and tumor T lymphocytes. A scatter plot with a linear regression line was used to show the relationship between HCC stiffness difference and overall survival time (or time to progression). All tests were two-sided and *p*-values of 0.05 or less were considered statistically significant. Statistical analyses were carried out using SAS version 9.3 and JMP version 14.0 (SAS Institute, Cary, NC).

## Results

Of the total of 15 patients, 4 withdrew (2 died before the followup MRE scan could be performed, 1 patient decided on hospice care and declined further treatment; 1 patient was intolerant to treatment), 1 patient did not undergo MRE scan, and 1 patient had MRE exam failure. The remaining 9 patients included 6 men. Median age was 71 years (range, 54–78).

HCC was well-differentiated in 2 of 9 patients, moderately differentiated in 6 and poorly differentiated in 1. Median overall survival and time to progression were 44 weeks (range, 16–70) and 13 weeks (range, 9–48), respectively. Etiology of liver disease was HCV (*n* = 4), HBV (*n* = 1) and NASH (*n* = 4).

No correlation was found for stiffness of non-tumor liver and etiology of liver disease.

Average non-tumor liver stiffness was 3.2 kPa (range, 2.1–4.3), and did not significantly change at 6 weeks (*p* = 0.42). Baseline non-tumor liver stiffness did not significantly correlate with overall survival, (*p* = 0.056), Table 1.

**Table 1:** Correlation of imaging and pathology with with overall survival; (R, Spearman correlation coefficient; kPa, kilopascals)

Variable	Average	Range	Correlation with Overall Survival (R)	*P-value*
Baseline HCC size (cm)	4.0	1.5–8.5	0.32	0.4
Change in HCC size (cm)	- 0.32	[− 2.2] - 0.4	0.21	0.58
Baseline HCC stiffness (kPa)	5.0	2.4–9.1	- 0.66	0.055
Change in HCC stiffness (kPa)	0.12	[− 2.1] – 2.8	**0.81**	**0.008**
Non-tumor liver stiffness (kPa)	3.2	2.1–4.3	- 0.65	0.056

Bold face type of numbers denotes findings are statistically significant

Seven of 9 HCC demonstrated > 50% enhancement at baseline, 1 demonstrated 20–50% and 1 < 25%. Decrease in HCC enhancement category was only seen in 2 of 9 patients at 6 weeks.

Correlation of overall survival with baseline and change in HCC size and stiffness are shown in Table 1. Average HCC size and change in size were 4 cm (range, 1.5–8.5) and - 0.32 (range, [− 2.2] - 0.4), respectively. There was no significant correlation between overall survival and baseline HCC size (*p* = 0.4).

**Fig. 1:**
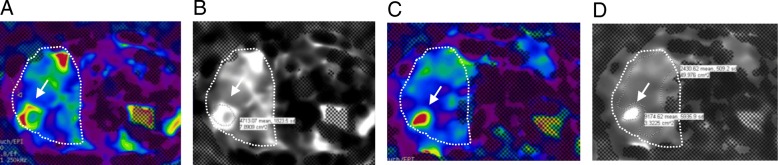
Elastogram color map. **a** gray scale **b** at baseline showing HCC (arrow). HCC stiffness increased on as indicated by increased red color of the tumor (**c** and **d**). Liver is demarcted by hashed lines (----)

**Fig. 2:**
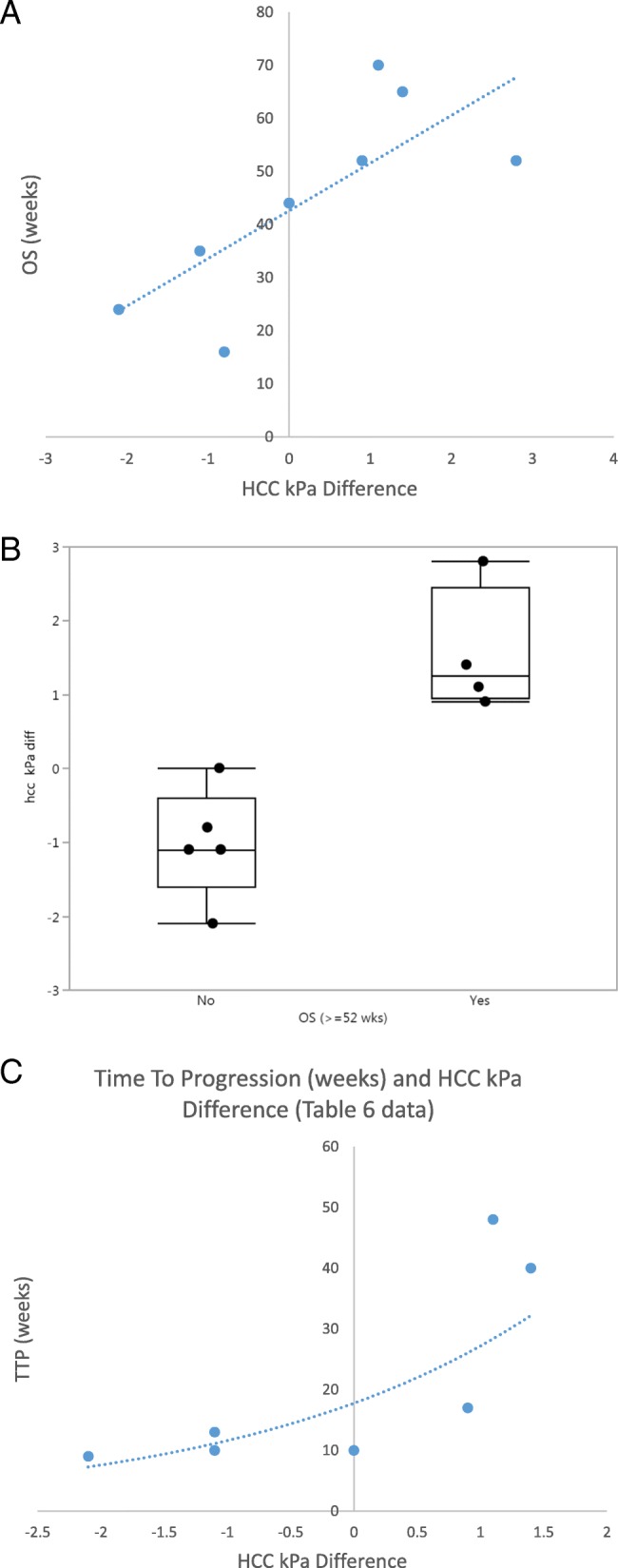
Association between MRE parameters and survival. **a** HCC stiffness difference (kilopascals, kPa) between baseline and 6 week MRE, correlated significantly with overall survival (OS), (Spearman *R* = 0.88, *p* < 0.05); **b** A greater increase in HCC stiffness (kilopascals, kPa) was significantly associated with a survival of more than 52 weeks from start of therapy, *p* = 0.02; **c** HCC stiffness difference (kPa) between baseline and 6 week MRE, correlated significantly with time-to-progression (weeks), (Spearman *R* = 0.88, *p* = 0.009; *n* = 7)

Average baseline HCC stiffness and change in stiffness were 5 kPa (range, 2.4–9.1) and 0.12 kPa (range, [− 2.1] – 2.8), respectively. There was no significant correlation between overall survival and baseline HCC stiffness (*p* = 0.055), Table 1. Increase in HCC stiffness on follow-up imaging (Fig. 1a and b) was seen in 5 patients, decrease in 3 patients and no change in 1 patient (Table 2). Increase in HCC stiffness at 6 weeks correlated significantly with overall survival (*R* = 0.81, *p* = 0.008), Fig. 2a, and with survival of more than 52 weeks from start of therapy (*p* = 0.02), Fig. 2b. Increase in HCC stiffness at 6 weeks also correlated significantly with time to progression (*R* = 0.88, *p* = 0.009), Fig. 2c.

**Table 2:** MRE HCC stiffness (kilopascals, kPa) at baseline and 6 weeks with overall survival (OS) and time-to-progression (TTP)

Patient	Baseline HCC stiffness (kPa)	6 week HCC stiffness (kPa)	Difference in stiffness (kPa)	OS (weeks)	TTP (weeks)
1	9.1	7	−2.1	24	9
2	2.8	3.9	1.1	70	48
3	7.0	6.2	−0.8	16	–
4	3.3	3.3	0	44	10
5	4.2	5.6	1.4	65	40
6	2.4	5.2	2.8	52	–
7	6.1	5.0	−1.1	35	13
8	6.7	7.6	0.9	52	17
9	3.8	2.7	−1.1	35	10

**Fig. 3:**
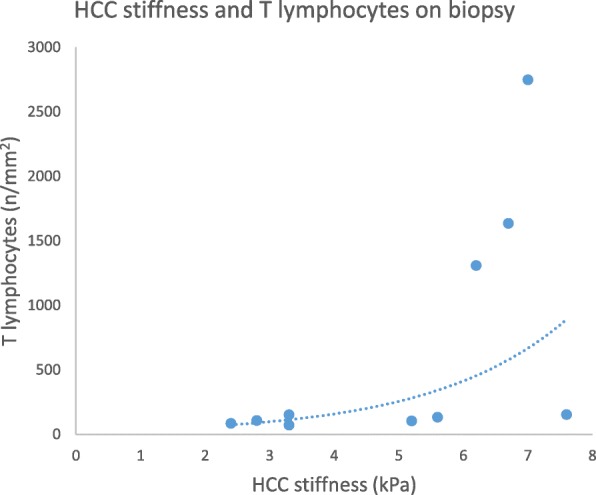
HCC stiffness correlated significantly with tumor T lymphocytes on biopsy (Spearman *R* = 0.79, *p* < 0.01)

HCC stiffness was significantly correlated with baseline HCC size (*R* = 0.7, *p* = 0.036), but not with tumor grade (*p* = 0.3). HCC stiffness was significantly correlated with tumor T lymphocytes (*R* = 0.79, *p* < 0.01) Fig. 3, however, pre-and post tumor lymphocyte evaluation was not available in all cases.

## Discussion

Imaging predictors of immunotherapy response in HCC could aid the identification of patients more likely to benefit from the treatment and represent an important unmet need. Our preliminary data suggests that an early increase in HCC stiffness may be an indicator of early immunotherapy (anti–PD-1) response.

There is limited data on MRE assessment of HCC. Thompson et al. [17], reported a trend toward increased HCC stiffness in well-moderately differentiated compared to poorly differentiated HCC but no correlation with size (*n* = 21). Our findings are concordant with this study since we also did not observe a significant correlation between tumor stiffness and tumor grade. However, this could have been due to there only being 1 patient with poorly differentiated HCC in our study. We observed a correlation between HCC stiffness and HCC size (*p* = 0.036), which is discordant with Thompson et al. [17]. This may have been related to differences in HCC stiffness measurement technique. Thompson et al. [17], only included solid portions of the tumor, but we included as much of the tumor as possible. Furthermore, it is possible that measurements of smaller tumors are assocated with partial volume averaging from the adjacent liver. Larger studies stratifying stiffness measurements with respect to tumor size are needed to understand the relationship between thesee observations.

To our knowledge, there are no prior studies evaluating HCC stiffness changes using MRE in patients treated with immunotherapy. Interestingly, increase in HCC stiffness at 6 weeks was correlated with improved outcome but not change in HCC size or enhancement. Our findings suggest effects related to immune cell infiltration and alteration in tumor stroma (including fibrosis and angiogenesis), can result in early increased tumor stiffness as an independant biomarker of response.

We found a significant correlation between HCC stiffness on MRE and the number of lymphocytes on tumor biopsy. This is supportive of the theory that anti-PD-1 therapy causes increased T lymphocyte activation in the immune-mediated response to tumor. However, further larger studies are necessary to better understand the underlying mechanisms. If our findings are confirmed, MRE would have an important clinical impact on the response assessment of advanced HCC treatment with checkpoint inhibitors, enabling early identification of treatment response.

Our study has some limitations. First, this is a pilot study that is meant to be hypothesis-generating, and the number of patients is small. Nevertheless, our study showed a strong correlation between early increase in tumor stiffness on MRE and overall survival. Further larger studies are necessary to validate our initial promising observations. Second, HCC was sampled with image-guide core biopsies. Given the heterogeneity of HCC, the biopsy sample may not have been representative of the whole tumor. However, we did observe a correlation between HCC stiffness and tumor T lymphocytes. Third, since patients were being treated with pembrolizumab, they had previously failed treatment or were intolerant to sorafenib. Prior treatment may have affected patient outcomes. However, anti-PD-1 therapy was used as a second-line treatment for all the subjects. Our study focused on change in HCC stiffness on serial MRE, and showed all patients that had an increase in tumor stiffness had better outcomes irrespective of baseline HCC stiffness. Fourth, in our exploratory study HCC stiffness measurements were made by a single independent radiologist placing region-of-interest on the tumor. Future larger studies could benefit having more than 1 radiologist measure tumor stiffness to allow assessment of interobserver agreement.

In summary, our preliminary data demonstrated that HCC stiffness increase on MRE in patients treated with immunotherapy significantly correlated with overall survival and time to progression. MRE has the potential to be a useful tool in the assessment of anti-PD-1 therapy in advanced HCC and may be beneficial to the many active immunotherapy trials.

## Conclusion

Our pilot MRE data suggests early change in tumor stiffness may be an indicator of immunotherapy response in patients with advanced HCC.

## Data Availability

The datasets used and/or analyzed during the current study are available from the corresponding author on reasonable request.

## Abbreviations

HCC: Hepatocellular carcinoma
MRE: Magnetic Resonance Elastograpy
